# Z-Scheme ZnO/ZnAl_2_O_4_ Heterojunction with Synergistic Effects for Enhanced Photocatalytic CO_2_ Reduction

**DOI:** 10.3390/molecules30122626

**Published:** 2025-06-17

**Authors:** Minhui Pan, Linlin Zheng, Congyu Cai, Weiwei Wang

**Affiliations:** College of Life Science and Chemistry, Minnan Science and Technology College, Quanzhou 362332, China; panminhui@mku.edu.cn (M.P.); 122592022020@mku.edu.cn (L.Z.); caicongyu@mku.edu.cn (C.C.)

**Keywords:** Z-scheme heterojunction, ZnO/ZnAl_2_O_4_ nanoparticle, zinc vacancies, oxygen vacancies, photocatalytic CO_2_ reduction

## Abstract

The photocatalytic reduction of CO_2_ into valuable hydrocarbons presents significant potential. In this research, a ZnO/ZnAl_2_O_4_ composite photocatalyst was synthesized using the hydrothermal method, resulting in a marked enhancement in CO yield—approximately three times greater than that achieved with pure ZnAl_2_O_4_ nanoparticles. The formation of a Z-scheme heterojunction between ZnO and ZnAl_2_O_4_ was observed, characterized by low interfacial charge transfer resistance, an abundance of reaction sites, and optimized charge transport pathways. Within this composite, ZnO contributes additional vacancies, thereby increasing active sites and enhancing the separation and migration of photogenerated carriers. In situ diffuse reflectance infrared Fourier transform spectroscopy (DRIFTS) analysis indicates that ZnAl_2_O_4_ facilitates the formation of key intermediates, such as *COOH and HCO_3_^−^, thus promoting the conversion of CO_2_ to CO. This study offers valuable insights into the design of heterogeneous catalysts with diverse active components to enhance the performance of CO_2_ photocatalytic reduction through synergistic effects.

## 1. Introduction

With the rapid growth of urbanization and industrialization brought about by global economic and technological advancement, human society has entered a new era. A number of ecological issues, including global warming, sea level rise, ocean acidification, faster permafrost thawing, and glacier retreat, have been brought on by excessive use of fossil fuels, which has increased CO_2_ emissions [1,2,3]. These issues pose a major threat to ecosystem stability and human health. As a result, reducing CO_2_ emissions has become an urgent global task, with efforts focused on sustainable development solutions [4,5]. Among the various solutions for mitigating the greenhouse effect, catalytic conversion of CO_2_ stands out for its lack of secondary pollution and high economic efficiency [6,7,8,9]. Because solar energy is inexhaustible and does not cause any pollution to the environment, photocatalytic CO_2_ reduction has a broad prospect [10,11,12]. A great deal of effort has been put in by researchers to find suitable materials to reduce CO_2_ into valuable products [13,14,15]. Out of all the photocatalyst possibilities, semiconductors are the most popular and promising [16,17,18].

ZnAl_2_O_4_ is a wide bandgap zinc-based spinel material with a forbidden bandwidth of about 3.8–3.9 eV, which shows better optical, electrical, magnetic, and catalytic properties under light excitation, and has been widely applied to various fields such as photocatalytic degradation of organic pollutants [19], sterilization and deodorization [20], catalytic reduction of CO_2_ [21], and so on. However, the wider bandgap structure greatly limits its application. The electrons and holes (e^−^, h^+^) generated by ZnAl_2_O_4_ are easily recombined, resulting in poor adsorption of CO_2_ and poor absorption of visible light. To solve these problems, researchers have tried various methods to modify it, such as elemental doping [22], morphology modulation [23], and construction of heterojunctions [24].

The creation of heterojunctions has proven to be an effective strategy for improving photocatalytic performance [25,26,27]. Composite materials can make up for the shortcomings of single materials, can not only maintain certain characteristics of single-phase catalyst components, but also through the composite structure to play a new performance. With a direct band gap of 3.2 eV, ZnO has been recognized as a high-quality material for photocatalytic processes due to its low synthesis cost, controllable morphology, and good thermal stability [28,29,30]. ZnO-based photocatalysts are even more effective than TiO_2_ in photodegradation applications [31]. ZnO and ZnAl_2_O_4_ are coupled to form a heterojunction through the energy band structure, which can induce the migration of photogenerated e^−^ and h^+^ between the conduction and valence bands and thus enhance the survival lifetime of photogenerated carriers [32]. It also increases the surface active sites of the material, which contributes to the photocatalyst reduction capacity [33,34].

In this study, we successfully synthesized ZnO/ZnAl_2_O_4_ composites via the hydrothermal method and employed them for photocatalytic CO_2_ reduction. Compared to individual ZnO and ZnAl_2_O_4_, the composites exhibited not only superior stability but also enhanced photocatalytic activity. The formation of Z-scheme heterojunctions facilitated the development of an internal electric field at the interface, which in turn promoted efficient space charge separation and provided high redox potentials for photogenerated carriers. The synergistic interaction between ZnO and ZnAl_2_O_4_ was elucidated through various characterization techniques. To further clarify the mechanism underlying the photocatalytic reduction of CO_2_, we utilized in situ DRIFTS to propose potential reaction pathways.

## 2. Results and Discussion

### 2.1. Morphology and Structural Characterization

**Figure 1:**
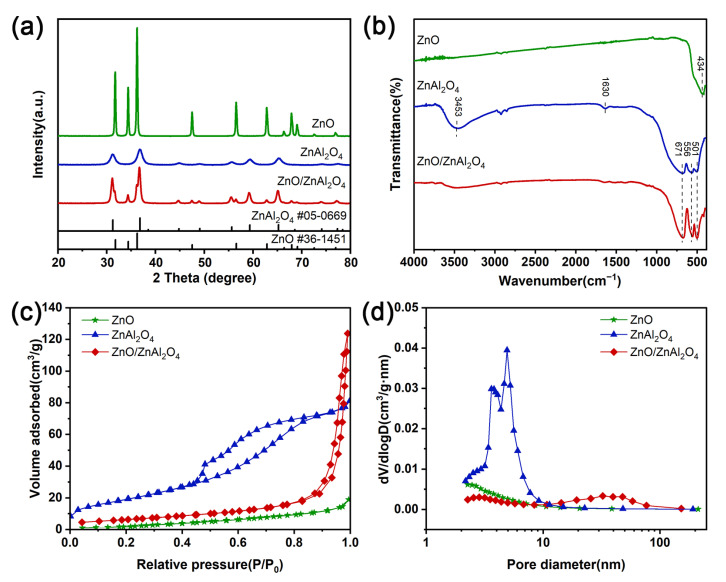
(**a**) XRD patterns, (**b**) FTIR spectra, (**c**) N_2_ adsorption–desorption isotherms, and (**d**) pore distribution of ZnO, ZnAl_2_O_4_, and ZnO/ZnAl_2_O_4_.

The XRD patterns of all the samples are shown in Figure 1a. The peaks of ZnO located at 31.8°, 34.4°, 36.3°, 47.6°, 56.6°, and 62.9° correspond to the (100), (002), (101), (102), (110), and (103) crystal planes of fibrillar zinc ore ZnO (JCPDS No. 36-1451). No other peaks were found, which proves the high purity of ZnO in the sample. And the sharp diffraction peaks indicate that the synthesized crystals are highly crystalline. The diffraction peaks of sample ZnAl_2_O_4_ at 31.2°, 36.8°, 59.3°, and 65.2° correspond to the (220), (311), (511), and (440) crystallographic planes of cubic ZnAl_2_O_4_ (JCPDS No. 05-0669). ZnO/ZnAl_2_O_4_ showed ZnO and ZnAl_2_O_4_ characteristic diffraction peaks, demonstrating the successful synthesis of the composite structure. It is noteworthy that the diffraction peaks of ZnO are significantly broadened and crystallinity is reduced after the composite, indicating a decrease in grain size or distortion of the lattice [35]. The disruption of the crystal lattice results in the formation of defects that increase the number of active sites on its surface, which in turn improves the catalytic performance.

The systematic molecular structure characterization of the samples was carried out using FTIR spectroscopy to resolve the functional group composition and chemical bonding structural features. As shown in Figure 1b shows that the absorption peaks at 3453 and 1630 cm^−1^ are caused by the stretching vibration of adsorbed water O-H or the bending vibration of adsorbed hydroxyl H-O-H on the surface of the sample material [20]. ZnO has a strong transmission peak near 434 cm^−1^, which is attributed to the stretching vibration of the Zn-O bond. The characteristic peaks at 671, 556, and 501 cm^−1^ can be attributed to Al-O stretching vibration, Al-O bending vibration, and Al-O asymmetric stretching vibration [36]. For the ZnO/ZnAl_2_O_4_ composites, the characteristic peaks of ZnO and ZnAl_2_O_4_ were observed in the low wavelength range of 400 to 1000 cm^−1^. Following the earlier XRD findings, the FTIR spectra thus show that the ZnO/ZnAl_2_O_4_ composite structure was successfully formed.

The samples’ N_2_ adsorption-desorption isotherms are displayed in Figure 1c. The mesoporous structure of each sample is demonstrated by type IV isotherms with H3-type hysteresis loops [37]. Figure 1d depicts the pore size distribution, and when combined with Table 1, it is clear that the addition of ZnO reduces the specific surface area of ZnAl_2_O_4_ while increasing pore volume and size. The smaller surface area of ZnO/ZnAl_2_O_4_ compared to ZnAl_2_O_4_ may be due to the fact that ZnO nanoparticles cover the surface and disordered pores are generated inside.

**Figure 2:**
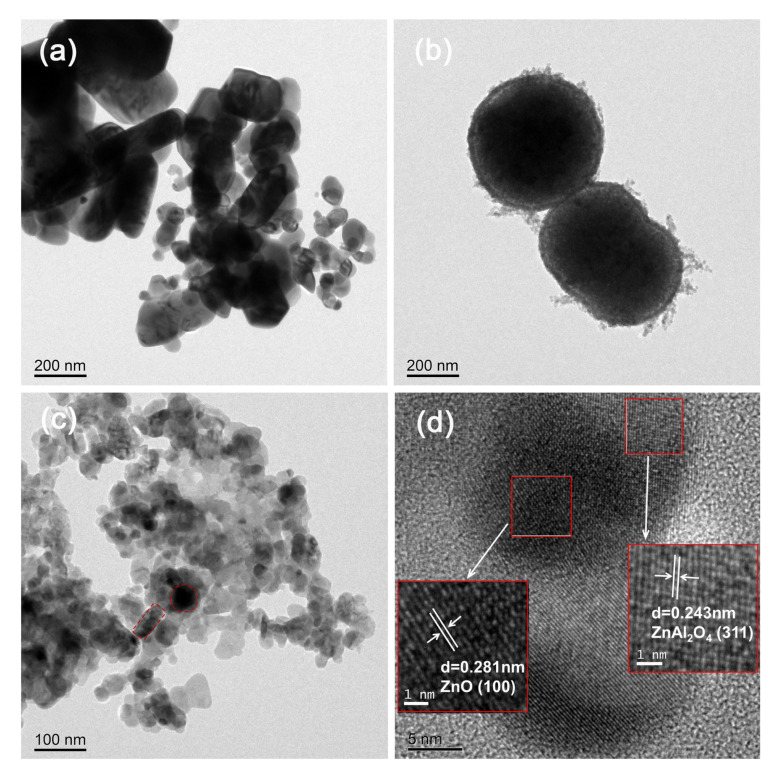
TEM images of ZnO (**a**), ZnAl_2_O_4_ (**b**), and ZnO/ZnAl_2_O_4_ (**c**); HRTEM image (**d**) of ZnO/ZnAl_2_O_4_.

TEM and HRTEM were used to study the microstructure of all samples. As can be seen from Figure 2a, ZnO behaves as irregular nanosquares with a wide range of lateral sizes, from 30 nm to 600 nm, whereas Figure 2b shows that ZnAl_2_O_4_ is a nano-microsphere structure with a sphere diameter of about 400–500 nm. Its edges are plush, increasing the contact area, so the specific surface area is the largest. Figure 2c shows that the circular ZnAl_2_O_4_ in ZnO/ZnAl_2_O_4_ is closely covered by massive ZnO nanoparticles with significantly reduced particle size. Their intimate contact makes it easier for charges to move between them and for the appropriate internal electric field to form [38]. Notably, no significant porosity was observed in the TEM images. This may be because the pore structure is not obvious or the TEM sample preparation process changed the pore structure. The HRTEM image of Figure 2d displays two distinct lattice fringes with lattice spacings of 0.281 and 0.243 nm, which correspond to the (100) and (311) crystal planes in ZnO and ZnAl_2_O_4_, respectively. This further confirms the successful preparation of ZnO/ZnAl_2_O_4_ heterojunction photocatalysts.

### 2.2. Surface Chemical States and Electron Distribution

**Figure 3:**
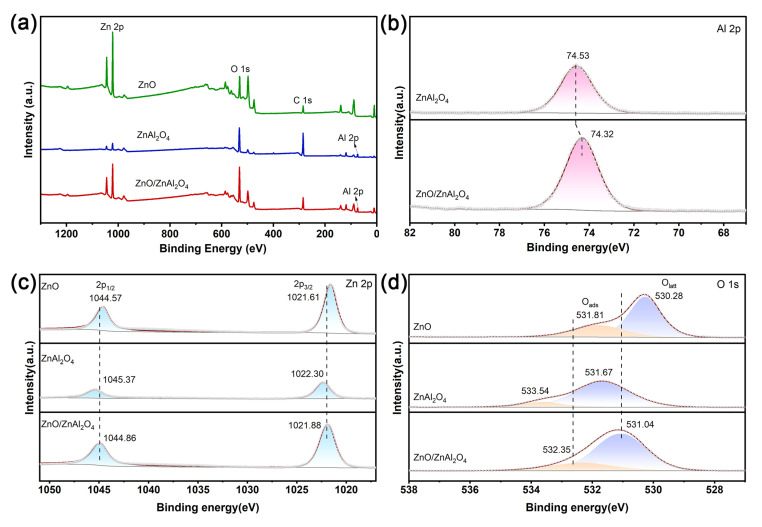
XPS of (**a**) survey spectra (**b**) Al 2*p*, (**c**) Zn 2*p*, and (**d**) O 1*s* for ZnO, ZnAl_2_O_4_, and ZnO/ZnAl_2_O_4_.

The combination of two materials has an unavoidable effect on the structure and charge state. The elemental composition and valence states of the samples are studied by X-ray photoelectron spectroscopy (XPS). The full spectrum (Figure 3a) shows that the prepared catalysts all contain Zn 2*p* and O 1*s* orbitals, indicating that they are all composed of Zn and O elements. Al 2*p* orbitals were also observed in ZnAl_2_O_4_ and ZnO/ZnAl_2_O_4_, which is consistent with the conclusions of the previous characterization. Figure 3b shows the detailed spectrum of Al 2*p* for different samples. From the figure, it can be seen that the Al 2*p* binding energy of ZnO/ZnAl_2_O_4_ is shifted towards a lower electric field compared to ZnAl_2_O_4_. These slight shifts prove a charge transfer between ZnO and ZnAl_2_O_4_, with ZnAl_2_O_4_ gaining electrons [39]. The material’s Zn valence state is +2, as indicated by the two different characteristic peaks in the Zn 2*p* XPS spectrum (Figure 3c), Zn 2*p*_3/2_ and Zn 2*p*_1/2_ [40]. In the ZnO/ZnAl_2_O_4_ heterostructure, there is a significant broadening of the FWHM of Zn 2*p*_3/2_ (1.55 eV for ZnO, 1.74 eV for ZnZnAl_2_O_4_, and 1.88 eV for the composite structure), which is due to the presence of Zn in two different chemical environments. The Zn 2*p* binding energy in ZnO/ZnAl_2_O_4_ composite photocatalysts is shifted to a higher energy direction compared to ZnO. And compared to ZnAl_2_O_4_, the binding energy is shifted to the low-energy direction. This again suggests the existence of strong interaction and charge transfer between ZnO and ZnAl_2_O_4_, as well as electron transfer from ZnO to ZnAl_2_O_4_ [41]. The O 1*s* XPS spectra (Figure 3d) show two morphologies of O, with the peak around 531 eV assigned to lattice oxygen (O_latt_) bonded to the metal, and the peak around 532 eV due to adsorbed oxygen (O_ads_) on the catalyst surface [42]. The decrease in binding energy of O 1*s* in ZnO/ZnAl_2_O_4_ is consistent with that of Zn 2*p*, demonstrating that the reaction process causes an increase in electron density, which promotes CO_2_ conversion. O_ads_ is commonly used to indicate the concentration of oxygen vacancies (O_v_) [43]. As can be seen from Table 2, the O_v_ concentration was enhanced from 13.20% to 21.95% after compounding with ZnO. The adsorption and activation of CO_2_ molecules, the provision of catalytically active sites for CO_2_ reduction, and the improvement of light energy use are all made possible by the presence of vacancies.

### 2.3. Optical and Photoelectrochemical Properties

**Figure 4:**
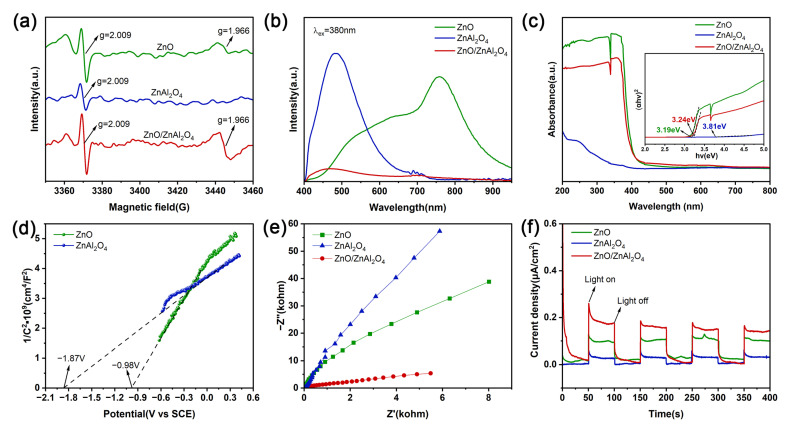
EPR spectra (**a**) and PL spectra (**b**) UV-vis DRS and Tauc plots (**c**) of ZnO, ZnAl_2_O_4_, and ZnO/ZnAl_2_O_4_; Mott–Schottky plots (**d**) of ZnO and ZnAl_2_O_4_; EIS Nyquist plots (**e**) and Transient photocurrent responses (**f**) of ZnO, ZnAl_2_O_4_, and ZnO/ZnAl_2_O_4_.

Sample vacancies were analyzed by electron paramagnetic resonance (EPR) spectroscopy. At the g = 2.009 position, a symmetric EPR signal appears for all specimens (Figure 4a). The symmetric signal corresponding to this value of g can be attributed to the trapped electrons on O_v_ [44]. The sharper EPR signal of ZnO/ZnAl_2_O_4_ compared to ZnO and ZnAl_2_O_4_ indicates a higher O_v_ content, suggesting that ZnO introduces vacancies again. A strong signal was also observed for ZnO and ZnO/ZnAl_2_O_4_ at g = 1.966, which is an indicator of zinc vacancies (Zn_v_) [45,46]. The separation and migration of photogenerated e^−^ and h^+^ are facilitated by more vacancies, which enhances the photocatalytic performance for CO_2_ reduction.

Using steady-state photoluminescence (PL) under 380 nm optical excitation, the complexation and separation characteristics of photogenerated e^−^ and h^+^ were examined (Figure 4b). ZnAl_2_O_4_ shows an emission peak at 480 nm, which is greatly attenuated when the complex is formed, implying that the formation of the complex inhibits the complexation of photogenerated carriers and favors the migration of photogenerated charges [47]. More photogenerated carriers are maintained when a heterojunction forms between ZnO and ZnAl_2_O_4_, passivating the non-radiative complex sites.

Samples were analyzed by UV-vis. As shown in Figure 4c, all three displayed significant light absorption in the UV spectrum. The corresponding forbidden bandwidths of the samples were calculated from Tauc plots [48]. The forbidden bandwidths of ZnO, ZnAl_2_O_4_, and ZnO/ZnAl_2_O_4_ are 3.19 eV, 3.81 eV, and 3.24 eV, respectively. It can be seen that the forbidden band range of the composite structure is mainly determined by ZnO, with enhanced light trapping compared to ZnAl_2_O_4_.

The location of the conduction zone was calculated by obtaining the Mott–Schottky (M-S) curve. The M-S curves for both ZnO and ZnAl_2_O_4_ have positive slopes, indicating that these samples are n-type semiconductors. For n-type semiconductors, the flat band potential is very close to the bottom of the conduction band position. Fitting the linear part yields slopes of 4.435 and 1.974 for ZnO and ZnAl_2_O_4_, respectively. Based on the slope = 2/Nqε_0_ε_r_, where q = 1.602 × 10^−19^ C, ε_0_ = 8.854 × 10^−12^ F/m, ZnOε_r_ ≈ 9, and ZnAl_2_O_4_ε_r_ ≈ 8.5, their charge carrier densities (N) can be estimated to be 3.533 × 10^22^ cm^−3^ and 8.404 × 10^22^ cm^−3^, respectively, which are tremendous values. In this case, the flat band potential can be approximated as the conduction band potential. The equivalent flat band potentials of ZnO and ZnAl_2_O_4_ are around −0.98 V and −1.87 V (vs. SCE), respectively, as Figure 4d illustrates. According to the calculation formula E_NHE_ = E_SCE_ + 0.244 V [49], the conduction band (CB) potentials of ZnO and ZnAl_2_O_4_ can be estimated as −0.74 V and −1.63 V (vs. NHE), respectively. Since E_VB_ = E_CB_ + E_g_ [50], ZnO and ZnAl_2_O_4_ have valence band (VB) potentials of 2.45 V and 2.18 V (vs. NHE), respectively. The CB and VB positions of the two are interleaved to form a Z-scheme heterojunction, which allows efficient electron transfer through the contact interface.

Interfacial charge transport resistance and carrier mobility were investigated using electrochemical impedance spectroscopy (EIS). The ZnO/ZnAl_2_O_4_ composites had the shortest Nyquist semicircle, according to the results, which are displayed in Figure 4e. This suggests that the combination of ZnO and ZnAl_2_O_4_ improves electron transfer kinetics and lowers interfacial charge transfer resistance [51]. The capacity of produced photocatalysts to separate charges has also been assessed using photochemical experiments. A higher charge separation efficiency is generally indicated by a stronger photocurrent response signal [52]. ZnO/ZnAl_2_O_4_ has the maximum photocurrent density and remains stable across four cycles of light and dark, as seen in Figure 4f.

### 2.4. Photocatalytic Performance

**Figure 5:**
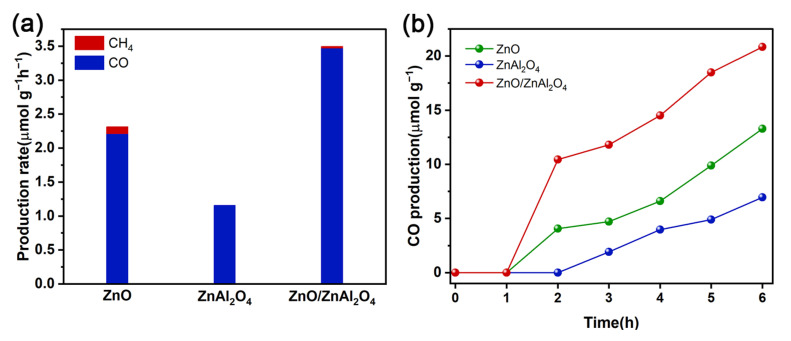
The production rate (**a**) and photocatalytic CO evolution performance (**b**) for CO_2_ reduction of ZnO, ZnAl_2_O_4_, and ZnO/ZnAl_2_O_4_.

The photocatalytic activities for CO_2_ reduction are measured in a solid-gas phase reaction consisting of CO_2_ and water vapor under a 300 W xenon lamp. As can be seen in Figure 5a, CO is the main product and ZnO and ZnO/ZnAl_2_O_4_ are accompanied by traces of CH_4_ by-products during the irradiation time. The optimal CO production rate of ZnO/ZnAl_2_O_4_ was 3.47 μmol g^−1^h^−1^, which was 1.57 times higher than that of ZnO (2.21 μmol g^−1^h^−1^) and 3.02 times higher than that of ZnAl_2_O_4_ (1.15 μmol g^−1^h^−1^). It can be found that after the formation of a heterojunction, the catalyst yield of CO is significantly increased. The main reason is that the layered structure of the complex can realize multiple light reflections. Meanwhile, the Z-scheme charge transfer can effectively inhibit carrier complexation and retain the strong redox capacity of photogenerated e^−^ and h^+^. The introduction of O_v_ and Zn_v_ in ZnO provides abundant active sites that enhance the adsorption and activation of CO_2_ molecules. The variation of CO production with reaction time was examined. Figure 5b shows that the CO production increased essentially linearly after 2 h of reaction. The CO yield of ZnO/ZnAl_2_O_4_ was significantly higher than the other two after 1 h of reaction and showed good stability.

### 2.5. Mechanism of Photocatalytic CO_2_ Reduction

**Figure 6:**
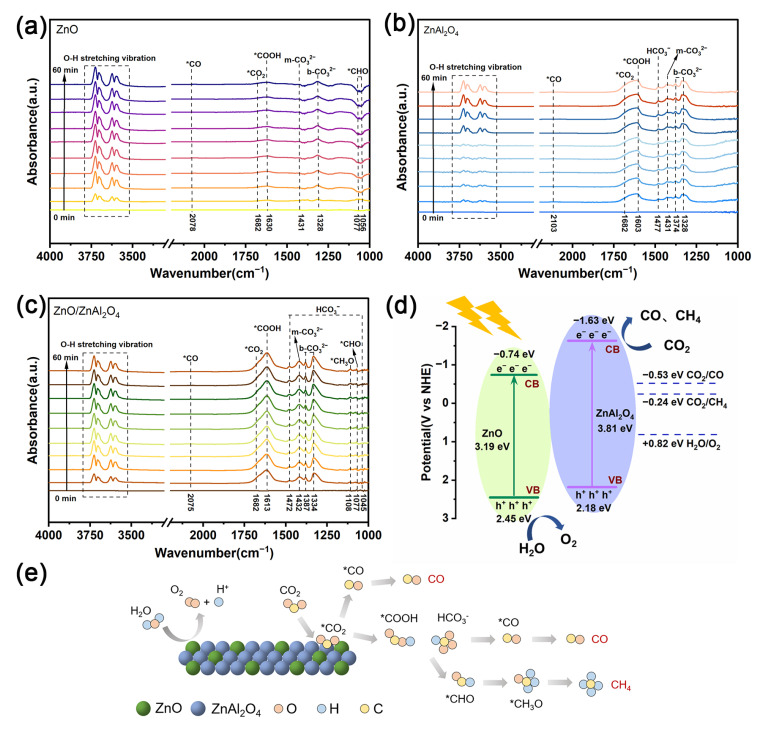
In situ DRIFTS spectra of CO_2_ reduction under light irradiation from 0 to 60 min for ZnO (**a**), ZnAl_2_O_4_ (**b**), and ZnO/ZnAl_2_O_4_ (**c**); (**d**) schematic illustration on the energy band structures and charge transfer pathways of ZnO/ZnAl_2_O_4_ under light irradiation; (**e**) the possible processes for CO_2_ reduction of ZnO/ZnAl_2_O_4_.

To identify important intermediates linked to the CO_2_ reduction reaction, in situ DRIFTS of the samples were obtained under light irradiation (Figure 6a–c). For all samples, the strong absorption peaks in the 3750–3550 cm^−1^ region were ascribed to both O-H stretching vibrations from H_2_O and adsorbed CO_2_ molecules [53]. Monodentate carbonate (m-CO_3_^2−^) was identified as the source of the apparent signals at 1432 and 1431 cm^−1^. The characteristic spectral bands at 1387, 1374, 1334, and 1328 cm^−1^ are attributed to bidentate carbonate (b-CO_3_^2−^) [41]. A characteristic peak of activated carbon dioxide molecules (*CO_2_) appears at 1682 cm^−1^ [54]. These peaks become more intense with time and their existence signifies that the adsorbed water molecules have dissociated and that the catalyst has been effectively activated by CO_2_. Signals for *COOH, an important intermediate in the conversion of CO_2_ to CO, were detected at 1630, 1613, and 1603 cm^−1^ [55]. It is noteworthy that the *COOH signals are stronger in ZnAl_2_O_4_ and ZnO/ZnAl_2_O_4_ compared to ZnO, and the characteristic peaks of bicarbonate (HCO_3_^−^) appear at 1472 and 1045 cm^−1^. ZnO and ZnO/ZnAl_2_O_4_ have weak signals of *CHO and *CH_3_O in the range of 1056–1108 cm^−1^, which leads them to generate small amounts of CH_4_ products [56].

The energy band structure and charge transfer diagram of ZnO/ZnAl_2_O_4_ can be created using the band gap and potential computation findings in 3.3 (Figure 6d). The misaligned energy band arrangement constitutes a Z-scheme heterojunction, which enhances the interfacial electric field, reduces the charge transfer resistance, and accelerates the charge separation. When exposed to visible light, e^−^ in ZnO and ZnAl_2_O_4_ VBs were stimulated into their corresponding CBs. The photogenerated electrons are then moved to the VB of ZnAl_2_O_4_ from the CB of ZnO in the heterojunction. This prolongs the lifetime of the photogenerated electrons and successfully prevents ZnAl_2_O_4_’s natural photogenerated e^−^ and h^+^ recombined. On the surfaces of ZnO and ZnAl_2_O_4_, H_2_O oxidation and CO_2_ reduction occur separately, realizing high redox potentials.

Three paths of CO_2_ reduction are presented in Figure 6e. The direct dissociation of CO_2_ is Path I. Following adsorption on the catalyst surface, CO_2_ transforms into *CO_2_. On the zinc surface’s carbon segment, the adsorption is more robust. The oxygen end binds to the proton and removes the O to produce *CO directly, and finally desorbs to produce CO [57]. In ZnO, CO_2_ reduction is mainly through this path. Path II is the formation of *CO_2_ after hydrogenation to form *COOH and HCO_3_^−^. On the catalyst surface, it breaks down into *CO and *OH, and *CO desorbs to create CO [58,59]. ZnAl_2_O_4_ provides a large amount of *COOH and HCO_3_^−^ intermediates for ZnO/ZnAl_2_O_4_ to promote the efficient desorption of CO. Path III is the continuation of the hydrogenation reaction of *CO to produce *CHO and *CH_3_O, and finally the formation of CH_4_ [60].

## 3. Experimental Section

### 3.1. Materials

The chemicals used in this work include zinc nitrate hexahydrate (Zn(NO_3_)_2_·6H_2_O, A.R.), aluminum nitrate nonahydrate (Al(NO_3_)_3_·9H_2_O, A.R.), ammonia (25% NH_3_·H_2_O, A.R.), and anhydrous ethanol (C_2_H_6_O, A.R.) and were purchased from Aladdin Reagent Co., Ltd. (Shanghai, China). In this experiment, all chemicals were utilized as received without any other treatment.

### 3.2. Synthesis of Photocatalysts

A specific amount of distilled water was used to dissolve Zn(NO_3_)_2_·6H_2_O and Al(NO_3_)_3_·9H_2_O in accordance with the Zn:Al = 1:2 ratio. Ammonia (25% NH_3_·H_2_O) solution was added dropwise to the mixture while being magnetically stirred until the pH reached 9.0. The reaction solution was moved to an autoclave lined with polytetrafluoroethylene after being stirred for 1 hour. After being sealed and maintained at 80 °C for 10 h, the autoclave naturally cooled to ambient temperature. Following many filter washes with ethanol and distilled water, the final sample was dried for a whole night at 80 °C in an oven. To achieve pure ZnAl_2_O_4_, the material was then crushed and calcined for 6 hours at 700 °C in a muffle furnace.

ZnO/ZnAl_2_O_4_ was prepared by a procedure similar to the one described above, with the difference that the amount of substances Zn/Al = 1:1. An excess of zinc nitrate was added to make the composite.

The same procedure was used to create pure ZnO for comparison; however, aluminum nitrate was not added.

### 3.3. Catalyst Characterization

The catalysts were characterized by X-ray diffraction (XRD) using a Rigaku MiniFlex600-C X-ray powder diffractometer at room temperature (Tokyo, Japan), Cu Kα, λ = 0.15418 nm, scanning range 20–80°. The catalysts were characterized by FTIR using a Shimadzu IRAffinity-1S Fourier infrared spectrometer (Kyoto, Japan), using the KBr pressing method. The catalysts were characterized by BET using a JW-BK200C specific surface area, pore volume, and pore size analyzer of Jingmicro Gobo Company (Beijing, China), the samples were treated in a vacuum at 300 °C for 8 h, and the nitrogen adsorption and desorption curves were obtained in a liquid nitrogen environment at −196 °C. The specific surface area of the samples was calculated using the multi-point BET method and the pore volume and pore size of the samples were analyzed using the BJH method. The morphological structure and grain size of the prepared samples were observed by a JEOL JEM-2100F transmission electron microscope (TEM) manufactured by Hitachi (Tokyo, Japan), and information such as lattice fringing of the samples was obtained by using the high magnification function of the microscope with an accelerating voltage of 200 kV. The catalysts were characterized by XPS using an ESCALAB 250 X-ray photoelectron spectrometer from Thermo Fisher (Waltham, MA, USA), and the binding energy was calibrated by the C1s peak of carbon (284.8 eV). The Electron Paramagnetic Resonance (EPR, BRUKER MS5000, Billerica, MA, USA) technique was used to detect and analyze free radicals or species with unpaired electrons in the samples. The photoluminescence (PL) characteristics of all the samples were obtained by a HORIBA Fluoromax-4 (Kyoto, Japan). The light response range of the sample was analyzed by UV-vis diffuse reflectance spectra (DRS) spectra (Shimadzu UV-3600, Kyoto, Japan) and Mott–Schottky (M-S) plots. Electrochemical impedance spectroscopy (EIS) and chronoamperometry (I-t) curves were measured on a CHI660E electrochemical workstation (Shanghai, China). The tests were carried out in a standard three-electrode configuration with the electrolyte as 0.1 mol/L Na_2_SO_4_ solution, the counter and reference electrodes as platinum and a saturated calomel electrode (SCE), respectively, and the working electrode as a thin ITO conductive glass coated with the catalyst sample. M-S curves were tested at 1000 Hz under light conditions. For the EIS test, the working electrode was tested over the frequency range of 0.1 to 100,000 Hz at 0 V (vs. SCE). The I-t curves were measured under the irradiation of a 300 W xenon lamp (PLS-SXE 300 C, Beijing Perfectlight, Beijing, China) at 0 V (vs. SCE), with the light source alternately turned on and off for 50 s during the test. For in situ DRIFTS, the dynamic evolution process was monitored using a Fourier in situ infrared spectrometer (Nicoletis10, Thermo Fisher, Waltham, MA, USA) at different time points. Prior to measurement, the samples were purged with N_2_ for 20 min in the sample chamber to eliminate surface contaminants and achieve stabilization. After collecting the background spectrum at t = 0 min, which was subsequently subtracted from all measurements, the gas source was switched to CO_2_: N_2_ mixture (2:8 *v*/*v*) with simultaneous light illumination (300 W xenon lamp). Samples were collected every 6 min for a total duration of 60 min.

### 3.4. Photocatalytic CO_2_ Reduction Reaction

The photocatalytic reduction of CO_2_ is carried out in a stainless steel reactor with a light-transmitting and high-pressure resistant quartz glass at the top, a heating device at the bottom, a thermocouple inside the reactor, and a sample stage for the catalyst. The light source for the photocatalytic reaction test was a 300W xenon lamp (PLS-SXE 300 C, Beijing Perfectlight, Beijing, China). For the experiment, 20 mg of catalyst powder was evenly spread in the reactor tray and placed in the reactor perpendicular to the beam. The temperature was raised to 120 °C. The reactor was sealed and leak tested to ensure the tightness of the reactor, then 0.3 mL of deionized water was injected into the reactor, the light source was turned on, and the reaction was carried out for 6 h. The gas product content was measured once every hour. The reaction products were analyzed using a gas chromatograph (GC9790II PLF-01, Ar as carrier gas) equipped with a thermal conductivity detector (TCD) and a hydrogen flame ionization detector (FID).

## 4. Conclusions

In this work, composite catalysts for the photocatalytic CO_2_ reduction reaction were successfully produced. Compared to individual ZnO and ZnAl_2_O_4_, the ZnO/ZnAl_2_O_4_ catalysts demonstrated higher photocatalytic performance under light. The Z-scheme heterojunction’s optimized charge-transfer pathways, increased CO_2_ reduction reaction sites, and decreased interfacial charge-transfer resistance were all credited with the activity boost. The separation and migration of photogenerated carriers were improved by the addition of ZnO, which greatly enhanced the quantity of oxygen vacancies (O_v_) and zinc vacancies (Zn_v_), which served as electron traps. And based on in situ DRIFTS, it was concluded that the addition of ZnAl_2_O_4_ enhanced the adsorption and activation of CO_2_, promoted the generation of the intermediate products *COOH and HCO_3_^−^, and significantly enhanced the CO yield. This study demonstrated that the synergistic effect of different active components could effectively promote the photocatalytic reduction performance of CO_2_.

## Figures and Tables

**Table 1:** Textural property of samples ZnO, ZnAl_2_O_4_, and ZnO/ZnAl_2_O_4_.

Samples	SBET (Specific Surface Area) m^2^/g	VP (Pore Volume) cm^3^/g	dP (Average Pore Size) nm
ZnO	10.71	0.03	10.38
ZnAl_2_O_4_	71.01	0.12	6.91
ZnO/ZnAl_2_O_4_	21.32	0.19	32.02

**Table 2:** The chemical state of Zn, Al, and O in the catalysts.

Samples	O Atomic/%		Atomic/%		
	O_latt_	O_ads_	Zn	Al	O
ZnO	68.02	31.98	47.55	0.00	52.45
ZnAl_2_O_4_	86.80	13.20	5.87	26.52	67.61
ZnO/ZnAl_2_O_4_	78.05	21.95	18.40	23.93	57.67

## Data Availability

Data are contained within the article.
